# Ankle brachial index as a surrogate to vascular imaging in evaluation of peripheral artery disease in patients with type 2 diabetes

**DOI:** 10.1186/s12872-020-01821-6

**Published:** 2021-01-06

**Authors:** Ejiofor Ugwu, Anthony Anyanwu, Michael Olamoyegun

**Affiliations:** 1 Department of Internal Medicine, Enugu State University of Science and Technology, Enugu, Nigeria; 2 Federal Medical Center, Owerri, Nigeria; 3 Ladoke Akintola University of Technology Teaching Hospital, Ogbomoso, Nigeria

**Keywords:** Ankle brachial index, Duplex ultrasonography, peripheral artery disease, Type 2 diabetes, Nigeria

## Abstract

**Background:**

Peripheral artery disease (PAD) is common in persons with type 2 diabetes (T2DM) and contributes significantly to cardiovascular morbidity and mortality. Controversy exists regarding the utility of ankle brachial index (ABI) for clinical diagnosis of PAD in persons with diabetes. The aim of this study was to evaluate the reliability of ABI for diagnosis of PAD in patients with T2DM using duplex ultrasonography (DUS) as the gold standard.

**Results:**

A total of 319 legs from 163 patients comprising of 156 subjects with intact legs and 7 patients who had undergone unilateral lower limb amputations were studied. The mean age of the participants was 56.1 ± 17.3 years. One hundred and ninety-five legs (61.1%) had sonographically confirmed PAD which was mild, moderate and severe in 40%, 41.5% and 18.5% respectively. The accuracy of ABI in detecting PAD was 76.7% for mild stenosis, 91.7% for moderate stenosis and 93.1% for severe stenosis. The sensitivity of ABI improved with increasing severity of arterial stenosis, reaching 100% in severe cases. ABI demonstrated good agreement with DUS [kappa = 0.65 (95% CI 0.49–0.88), *P* < 0.001].

**Conclusion:**

In comparison to DUS, the ABI demonstrated good reliability for diagnosis of PAD in high risk T2DM patients. The utility of this simple and non-invasive procedure should therefore be maximized in clinical practice.

## Background

Peripheral artery disease (PAD) refers to atherosclerotic occlusive disease of the systemic arteries excluding the coronary arteries. Lower extremity PAD is a marker of systemic atherosclerosis and an independent risk factor for major cardiovascular events including stroke, myocardial infarction and cardiovascular death [1–3]. The risk factors of PAD are those of cardiovascular diseases generally and include older age, hypertension, diabetes mellitus, cigarette smoking and hypercholesterolemia [4].

Individuals with type 2 diabetes mellitus (T2DM) are three times more likely to develop PAD than those without [5]. Lower limb PAD is reported in 35–60% of T2DM patients with diabetic foot ulcer (DFU) and contributes significantly to its development, progression and outcome [6]. Lower limb PAD delays healing of diabetic foot wounds by several mechanisms including limitation of nutrient supply to the tissues and impairment of antibiotic delivery to infected wounds [7, 8]. In fact, lower limb PAD is an independent risk factor for lower extremity amputation (LEA) in patients with DFU [6, 9]. Foot ulcer recurrence is reportedly five times more likely in diabetic patients with PAD than those without [6]. Peripheral artery disease is therefore an important morbidity requiring serious attention in persons living with diabetes.

The gold standard for diagnosis of PAD used to be digital subtraction angiography (DSA) but this has been replaced with computed tomography angiography (CTA) or magnetic resonance angiography (MRA) due to the invasive nature and potential risks associated with the procedure [10]. Nevertheless, CTA and MRA carry the risks of ionizing radiation and contrast associated toxicity respectively, in addition to being expensive. Duplex ultrasonography (DUS) has the advantage of being non invasive, does not involve use of contrast or ionizing radiation, and therefore safer. In spite of being largely operator-dependent, the reliability of DUS for detection of PAD has been demonstrated in several settings [11, 12]. The National Institute for Health and Care Excellence (NICE) recommends DUS as the first line imaging modality for PAD while reserving angiography for those who require further evaluation [13]. Despite the relative advantages of DUS over angiography, cost, the sophistication of the equipment and need for specialized personnel limit its application in routine clinical practice. This is even more so in low resource settings such as developing countries with poorly equipped healthcare systems.

The ankle brachial index (ABI) which is a ratio of the highest ankle systolic blood pressure obtained in the anterior tibial/dorsalis pedis or posterior tibial artery to that of the highest brachial systolic pressure is the most widely screening tool for lower limb PAD in clinical practice. An ABI less than 0.9 is generally regarded as indicative of lower extremity PAD [14, 15]. In comparison to angiography and DUS, ABI measurement uses simple readily available and affordable equipment, making it a more attractive and valuable option for evaluating PAD. Besides, it has demonstrated acceptable reliability in many populations [14, 16]. However, there are evidences suggesting that the ABI may not be a reliable tool for assessing PAD in subjects with diabetes [14, 17]. This study was aimed at evaluating the diagnostic accuracy of ABI for lower limb PAD in subjects with T2DM.

## Methods

### Study design and population

This was a cross-sectional study conducted at the medical out-patient department of the Enugu State University Teaching hospital between June 2017 and August 2018. The inclusion criteria were subjects aged 30–80 years, who had been diagnosed with T2DM according to the 1999 World Health Organization criteria [18], and who have clinical suspicion of lower extremity PAD based on history of intermittent claudication (recurrent exertional calf pains that are relieved by rest) and/or diminished/absent peripheral pulses (dorsalis pedis or posterior tibial) on manual palpation. Subjects with active DFU and those with pedal edema that extended above the ankle joint were excluded from the study. The Research and Ethics Committee of Enugu State University Teaching Hospital approved the study protocol while informed consent was obtained from each patient prior to recruitment.

### Sample size determination

The minimum sample size for the study was determined by Fisher’s formula: N = Z^2^PQ/d^2^ where N = minimum sample size, Z = the standard normal deviate corresponding to a 95% confidence level (I.e. 1.96). P = prevalence of peripheral artery disease in subjects with T2DM i, e, 12% [19], Q = complementary probability i.e., 1 − P = 1 − 0.12 = 0.88, and d = absolute precision limit desired (5%) = 0.05. Thus N = (1.96)^2^ (0.12) (0.88)/(0.05)^2 ^≈ 162. Subjects were consecutively recruited on each diabetes out-patient clinic day until a sample size of 170 was attained.

### Initial screening and clinical examinations

After obtaining demographic and diabetes related history, history of intermittent claudication was sought. This was followed by manual palpation of the dorsalis pedis and posterior tibial artery pulsations in both lower limbs. Subjects were recruited for further vascular assessments (ABI and DUS) if they admitted to positive history of intermittent claudication and/or were adjudged to have diminished or absent peripheral pulses in at least one lower limb.

### Ankle brachial index measurements

Oscillometric method using digital automatic blood pressure apparatus (OmronMX2,Omron Healthcare Europe B.V. Hoofddorp, The Netherlands) was used to obtain brachial and ankle (posterior tibial) blood pressures in both upper and lower extremities. Vegas et al. [16] had demonstrated that this method was more accurate than using manual blood pressure device and a Doppler probe. Examination was done with the patient lying supine after at least a 15-min rest. The ABI for each leg was calculated as a ratio of ankle blood pressure to that of the mean arm pressure. A ratio in the range 0.9–1.3 was regarded as normal according to international recommendations [13–15]. An ABI < 0.9 was diagnostic of PAD and values were stratified according to severity as follows: 0.7–0.89 = mild arterial obstruction, 0.5–0.69 = moderate arterial obstruction, ≤ 0.5 = severe arterial obstruction [14]. Subjects with ABI > 1.3 were adjudged to have incompressible lower limb arteries and excluded from analysis.

### Duplex ultrasonography assessment

Duplex ultrasonography of the lower extremity arteries, from common femoral to pedal arteries was performed for each patient not later than two weeks following ABI measurements using a DC 60 Colour Duplex Scanner and a 7.5 MHz linear-array transducer (Mindray, China). The test was conducted by a certified radiologist and followed standard imaging protocols. Examination was done with the patient lying supine after resting for at least 15 min. The common femoral and anterior tibial arteries were imaged with the patient in the supine position while those of the popliteal, peroneal and posterior tibial arteries were done in lateral position. Imaging views were taken parallel to the vessel walls and within the center of laminar flow while maintaining the Doppler at or below angle 60°. Identification of arterial segments was based on the appearance of color signals, and where the artery is occluded, by the detection of a vessel wall accompanied by a vein. When turbulence or velocity increase was detected in the color image or the B-mode image suggested a change in vessel caliber, then flow velocity measurements were performed by means of spectral analysis and peak systolic velocities (PSV) recorded in meters per second (m/s).

Peripheral artery disease was diagnosed by the presence of at least a 50% reduction in luminal diameter, corresponding to PSV ≥ 150 cm/s. The severity of stenosis was graded as follows: 50–75% (PSV 200–300 cm/s) = mild stenosis; 76–99% (PSV > 300 cm/s) = moderate stenosis; complete occlusion = severe stenosis [20]. Complete luminal occlusion was diagnosed by the following criteria: loss of signal in the vessel segment, attenuated distal signal relative to that of the proximal, or detection of proximal exit or distal re-entry collaterals.

### Data analysis

Data were analyzed with the Statistical Package for Social Sciences software (IBM version 23.0; SPSS Inc., Chicago, IL, USA). Categorical variables were presented as numbers and percentages while continuous variables were presented as means and standard deviations. Data were presented in tables and charts as appropriate. The sensitivity, specificity, positive predictive value, negative predictive value and overall accuracy of ABI for diagnosis of PAD based on DUS as the gold standard were computed. Analysis was done at limb level rather than participants since ABI was calculated separately for each limb in line with international guidelines [13, 15]. Agreement between ABI and DUS was tested by Cohen’s Kappa (*κ*) statistics. The following stratification of *κ* was used: *κ* value of 1 = perfect agreement, *κ* 0.8–0.99 = excellent agreement, *κ* 0.6–0.79 = good agreement, *κ* 0.4–0.59 = moderate agreement, *κ* < 0.4 = poor agreement and *κ* value of zero signifies no agreement beyond chance.

## Results

Out of the 170 subjects recruited, 7 were excluded due to arterial stiffness resulting in ABI > 1.3. In total, 319 legs from 163 patients comprising of 156 subjects with both legs intact and 7 patients who had undergone unilateral lower limb amputations were studied. They were 46.6% males and over 80% were above 45 years of age. Cigarette smoking was uncommon in the study population while 58.9% suffered from systemic hypertension. Table 1 summarizes the demographic characteristics of the study population.

**Table 1:** Demographic profile of the study population

Variable	Value
Gender	
Male	76 (46.6)
Female	87 (53.4)
Age (years)	56.1 ± 17.3
< 45 years	23 (14.1)
45–64 years	99 (60.7)
≥ 65 years	41 (25.2)
Cigarette smoking	
Yes	10 (6.1)
No	153 (93.9)
Diabetes duration (years)	8.6 ± 5.9
≤ 10 years	114 (69.9)
11–20 years	45 (27.6)
> 20 years	4 (2.5)
Intermittent claudication	
Yes	55 (33.7)
No	108 (66.3)
Hypertension	
Yes	96 (58.9)
No	67 (41.1)
Previous stroke	
Yes	18 (11.1)
No	145 (88.9)
Previous foot ulcer	
Yes	31 (19.0)
No	132 (81.0)
Previous amputation	
Yes	7 (4.3)
No	156 (95.7)

Data are in n (%) or mean ± SD

Table 2 shows the prevalence and severities of sonographically confirmed lower limb PAD. A total of 195 legs (61.1%) had sonographically confirmed PAD which was mild, moderate and severe in 40.0%, 41.5% and 18.5% respectively. The ABI demonstrated a low sensitivity of 54% for mild arterial stenosis, although with high specificity of 91%. The sensitivity of ABI improved with increasing severity of arterial stenosis, reaching 100% in severe cases. Similar trend was observed in the overall accuracy of ABI in detecting PAD which was 76.7% for mild stenosis, 91.7% for moderate stenosis and 93.1% for severe stenosis.

**Table 2:** Sensitivity, specificity, positive predictive value, negative predictive value and accuracy of the ankle brachial index at varying degrees of arterial occlusion

ABI	Sonographic stenosis		Sensitivity (%)	Specificity (%)	PPV (%)	NPV (%)	Accuracy (%)
	**Mild**	**None**					
PAD	42	11	54	91	79	76	76.7
Normal	36	113					
	**Moderate**	**None**					
PAD	75	11	93	91	87	95	91.7
Normal	6	113					
	**Severe**	**None**					
PAD	36	11	100	91	77	100	93.1
Normal	0	113					

ABI, ankle brachial index; PAD, peripheral artery disease; PPV, positive predictive value; NPV, negative predictive value

**Fig. 1:**
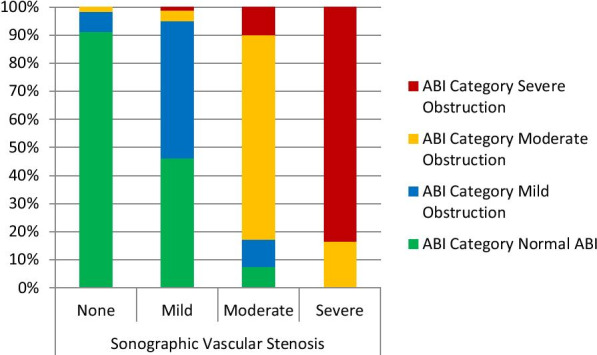
Agreement between duplex ultrasonography and ankle brachial index. Cohen’s kappa (K) = 0.65 (95% CI 0.49–0.88), *P* < 0.001. ABI = Ankle Brachial Index

**Fig. 2:**
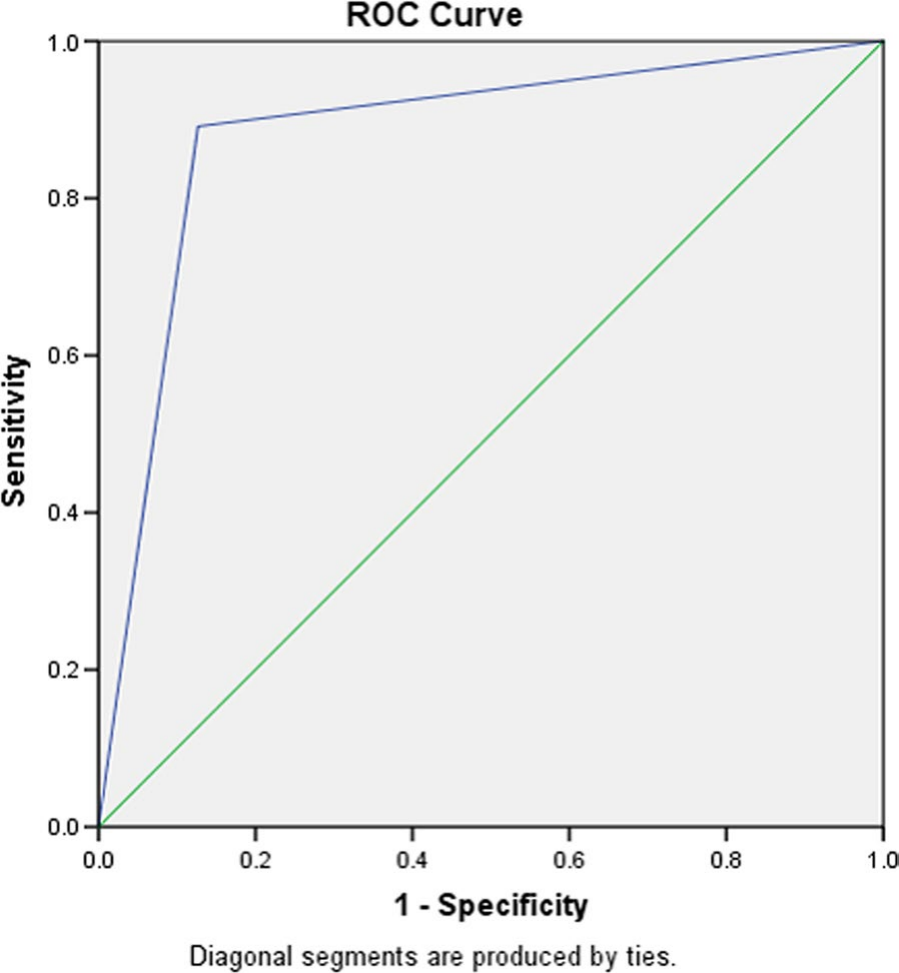
Receiver operating characteristics curve for ankle brachial index versus duplex ultrasonography AUC = 0.88 (95% CI = 0.82–0.94)

Agreement between ABI and DUS at varying severities of arterial obstruction is presented in Fig. 1. Ankle brachial index demonstrated good agreement with DUS [kappa = 0.65 (95% CI 0.49–0.88), *P* < 0.001] (Fig. 2).

## Discussion

Findings from this study affirm the utility of the ankle brachial index as a reliable diagnostic tool for peripheral artery disease in subjects with type 2 diabetes mellitus. The ABI demonstrated good diagnostic accuracy and good agreement with duplex ultrasonography in this high risk population of T2DM subjects. This observation is reassuring in the light of several published studies calling for caution in the use of ABI for diagnosis of PAD in people with diabetes [13, 14, 17]. In the year 2012, and updated in 2018, the National Institute for Health and Care Excellence specifically stated that diagnosis of PAD should not be ruled out in people with diabetes on the basis of a normal ABI, pointing out that such people are more prone to arterial calcification and therefore may have falsely elevated ankle pressures [13]. Our findings however do not seem to uphold such view. Instead, our observations corroborate those of other researchers who had found the ABI to be reasonably accurate for assessing lower limb PAD in people with diabetes [21-23]. In fact, in one study in United Kingdom that evaluated the accuracy of ABI in subjects with T2DM, ABI had a strong correlation, and sensitivity of 80% and specificity of 93% when compared with duplex angiography [23]. Our observations are not significantly different from those reported in non-diabetic populations in whom ABI demonstrated reliable accuracy in comparison with DUS and angiography [14, 16].

Compared to DUS however, we observed that ABI demonstrated low sensitivity in subjects with mild arterial obstruction and therefore failed to identify individuals with early disease in whom it missed nearly half of the cases. The implication of this finding in clinical practice is that in the event of a compelling need to establish the presence of PAD in type 2 diabetic patients with clinical suspicion of early disease, vascular imaging rather than ABI should be the modality of choice. On the other hand, in subjects with moderate to severe disease, the ABI demonstrated both high sensitivity and specificity, and good overall accuracy. In fact, in those with severe arterial obstruction on DUS, ABI demonstrated a sensitivity of 100% and showed high specificity as well as high positive and negative likelihood ratios.

Although imaging modalities such as DUS, CTA and MRA remain the gold standard for diagnosis of PAD, and especially for anatomic delineation of vascular stenosis, these diagnostic techniques are expensive and require highly skilled manpower. Consequently, they are not widely available, more so in resource-constrained settings as obtainable in most developing countries. Yet, these developing countries are experiencing rapid increases in the prevalence of diabetes with resultant increase in the burden of diabetes complications [24]. The ABI on the other hand uses inexpensive devices that are readily available and require minimal training to operate. It is therefore heart-warming to observe that this simple technique demonstrated reasonable accuracy for diagnosis of PAD in the diabetic population similar to its performance among subjects without diabetes. It is noteworthy that the American Diabetes Association recommends that ABI should be measured in all diabetic patients older than 50 years of age as well as those symptomatic of PAD or other cardiovascular risk factors [25]. Nevertheless, the limitations of ABI in clinical practice need to be emphasized. For instance, ABI measurement is time consuming and may be fraught with inconvenience. It is also associated with technical difficulties in subjects with leg edema or large foot wounds. Importantly, the ABI is unable to detect the exact point of arterial stenosis, and may present false negative results in subjects with vascular calcifications such as elderly patients and patients with advanced renal diseases.

The limitations of our study deserve a highlight. Firstly, this study was conducted among T2DM subjects with strong clinical suspicion of PAD. Therefore, our findings may not apply to the general population of diabetic subjects such as those typically encountered in routine clinical practice. Secondly, the Doppler probe method of ABI measurement remains more popular in clinical practice than the oscillometric method that was used in this study, despite evidences that the latter is probably more accurate. Caution should therefore be exercised in generalizing the findings from this study to include the Doppler method. Lastly, this study was conducted in a single center and among a relatively small population of diabetic subjects. Our findings may therefore not be generalized to the entire population of T2DM subjects.

## Conclusion

This study demonstrated that the ankle brachial index is reasonably accurate and reliable for the evaluation of peripheral artery disease in subjects with type 2 diabetes, and demonstrated good agreement with duplex ultrasonography method. However, clinicians need to be mindful of its low sensitivity in subjects with mild disease which may result in false negative tests. Bearing this as well as other known limitations of ABI in mind, the utility of this simple, non invasive cheap and readily available peripheral artery disease assessment tool can be maximized, particularly in developing countries where vascular imaging, the gold standard is not readily available.

## Data Availability

The datasets used and/or analyzed during the current study are available from the corresponding author on reasonable request.

## Abbreviations

ABI: Ankle brachial index
CTA: Computed tomography angiography
DFU: Diabetic foot ulcer
DSA: Digital subtraction angiography
DUS: Duplex ultrasonography
MRA: Magnetic resonance angiography
NICE: National Institute for Health and Care Excellence
NPV: Negative predictive value
PAD: Peripheral artery disease
PPV: Positive predictive value
PSV: Peak systolic velocity
T2DM: Type 2 diabetes mellitus
